# Piaget as scientific diplomat: Exchanges between the Geneva School and Soviet psychologists during the 1950s–60s

**DOI:** 10.1177/09526951251343796

**Published:** 2025-06-18

**Authors:** Luciano Nicolás García, Ramiro Tau, Marc J. Ratcliff

**Affiliations:** 162196 Sigmund Freud PrivatUniversität, Austria; Consejo Nacional de Investigaciones Científicas y Técnicas, Argentina; 27212 Université de Genève, Switzerland; 27212 Université de Genève, Switzerland

**Keywords:** Cold War, developmental psychology, International Congresses, politics, scientific diplomacy

## Abstract

It is known that Jean Piaget visited the Union of Soviet Socialist Republics and other socialist countries during the second half of the 20th century. However, these trips are usually mentioned in historical works as one more episode referring to Piaget’s international circulations or as a marginal information in the general plan of discussing divergences, complementarities, or incompatibilities between Lev Vygotsky’s psychology and Piagetian genetic psychology. In this article we intend to provide some arguments and archival evidence to open new avenues of reflection on the links between the Geneva School and Soviet psychology, based on the exchanges that Piaget and his Geneva research team had with several Soviet colleagues between 1954 and 1966. For this purpose, we have analysed some unpublished material from the documentary collection of the Centre Jean Piaget at the University of Geneva. In particular, the personal and institutional correspondence between the Swiss and Russian teams, promoted around Piaget’s visits to Moscow and Leningrad in 1955 and 1966. These documents show Piaget’s efforts to engage in scientific diplomacy in order to articulate his research programme, with his institutional positions and the international exchanges, while navigating the political tensions of the Cold War.

Jean Piaget is one of those figures who their celebrity earned though his publications has eclipsed some other basic aspects of his work. Typically, literature on Piaget has focused on theoretical and methodological aspects (Ratcliff, 2024), understandably so as they were relevant contributions to psychology and other disciplines, but not much in the institutional underpinnings and organization of work, both aspects that were conditions of possibility and extensions of his research programme. More recently, historical enquiries have tries to reinstate this dimension of Piaget’s work and persona (e.g. Hofstetter and Schneuwly, 2022; Ratcliff and Burman, 2017; Ratcliff and Tau, 2019), showing new aspects of his thinking, organizational skills, and political standing. This latter aspect is relevant to tackle due to a series of factors: Piaget’s longevity and production during many political changes in Europe; his positions in international institutions such as the United Nations Educational, Scientific and Cultural Organization (UNESCO), the International Union of Psychological Science (IUPSYS), and the International Bureau of Education; and his active and constant involvement with researchers from all over the world through the Centre international d’épistémologie génétique (International Centre of Genetic Epistemology, CIEG).

While there is increasing interest in Piaget’s travels, connections, and academic reception throughout the Americas and Western Europe (e.g. Caruso and Fairstein, 2003; Freitas Campos, Lourenço, and Ratcliff, 2023; Hsueh, 2009; Jaccard, 2020), his relationship with Eastern European scholars and political matters is still largely overlooked (van der Veer, 1996a), despite the fact that, according to preliminary information from the Jean Piaget Archives, he crossed the ‘Iron Curtain’ no less than seven times between 1955 and 1971. The relationship between Piaget and psychologists from the socialist bloc during the Cold War era is very informative in respect to the position of the Swiss scholar as an international reference, and the ways Piaget thought to advance, not only his research programme, but Psychology as discipline. Certainly, travels to the socialist bloc were almost never about ‘pure’ and ‘uninterested’ scientific matters – an untenable stance when science became a key aspect of international tensions to begin with (e.g. Oreskes and Krige, 2014; Turchetti, 2020). Because of this, and the institutional involvement of Piaget already mentioned, it is possible to consider Piaget’s endeavours in terms of *scientific diplomacy*, especially in regards with his travels to the Union of Soviet Socialist Republics (USSR). This approach, however, requires certain clarifications.

Contrary to a stricter definition based in institutional authorities positioning science as part of broader economic and political arrangements (e.g. Ruffini, 2017; Turekian, 2018), a broader notion of science diplomacy can account for ‘an array of different interactions taking place within the global politics-science interphase and, more importantly, as a heuristic tool to navigate and distinguish between different types of interactions’ (Kaltofen and Acuto, 2018: 10). Literature on the topic has distinguished between science *in* diplomacy – scientific expertise and data relevant to diplomatic action – diplomacy *for* science – that is, the governmental facilitation of international science cooperation – and science *for* diplomacy – science cooperation for improving international relations. Yet, these distinctions do not consider the production of knowledge as a core scientific aim. As Adamson and Lalli (2021) noted, the missing distinction, diplomacy *in* science, would be a better formula to understand how researchers navigate political differences while trying to promote productive exchanges. The focus can be then shifted to the initiatives of the scientists, consider the actual research results, and the nuances typical of interactions that try to balance political and/or scientific positions that can be antagonistic. In this view, the circulation of knowledge and the international construction and reproduction of a discipline can also be regarded as a diplomatic effort in itself, considering that political differences and open conflicts among several countries have been a constant around the world. For the case of this article, science diplomacy can be regarded as institutional actions and intellectual dialogues directed towards strengthening a research agenda in cases where political and national interests do not necessarily align. While researchers always represent, one way or another, national identities and political stances, and their institutions might be closely tied to the state, government authorities are not always the main promoters nor mandatory mediators when it comes to scientific networking. Moreover, the formal endorsement of state institutions does not imply that authorities control or direct the objective of researchers; conversely, many times researchers have tried to promote their work against the interests of their current national authorities. Yet highlighting the autonomy of scientific disciplines does not mean that they can be indifferent to geopolitical dynamics or political sensibilities, especially when talking about the Cold War conjuncture. The point is that the junctions between political dynamics and disciplinary autonomy need to be historically reconstructed, politics and policies are not a monopoly of government authorities, and ideologies cannot be considered a priori a facilitation or an obstacle for scientific organization.

As mentioned, by the 1950s Piaget held an important and visible positions internationally, yet this role has been overshadowed by his theoretical production, which is presented in a typical aseptic fashion, a self-sufficient individual researcher whose most important feature is his intellectual prowess. This is typically illustrated by the way his ideas have been opposed to those of Lev S. Vygotsky, another author who has been analysed in a highly decontextualized manner (García, 2016; Van der Veer, 2007; Yasnitsky and Ferrari, 2008). Therefore, the exchanges of the researchers at Geneva with the Soviet counterparts were known but only as purely theoretical comparisons between the two leading theorists, under the assumption that a lack of communication led to essentially different approaches. There is a substantial amount of literature devoted to either find or propose theoretical or methodological convergences between their two approaches on child development (Castorina and Baquero, 2005; Duncan, 1995; Forman and Kraker, 1985; Tudge and Rogoff, 1989; Veraksa and Samuelsson, 2022) or the recognition of irreducible incompatibilities (Bakhurst *et al.*, 1988; Bruner, 1997; Hyman, 2012; Smith, Dockrell, and Tomlinson, 2003; Tryphon and Vonèche, 1996). Undoubtedly, theoretical comparisons can be productive for research, but they do not offer a proper historical account on how real interaction among authors were made, or how such exchanges play a part in the building of a disciplinary field. In any case, the narratives of a failed meeting, mutual incomprehension, or two essentially disconnected approaches have not been contrasted with historical evidence nor with a perspective that would consider Piaget activities beyond his canonical image as a sole grand theorizer. Thus, this article will not focus on Piaget’s theoretical propositions or debates, but as international organizer of psychology as a scientific discipline, as this dimension of his work has been usually relegated, yet it sheds light on important aspects of his intellectual itinerary and scholarly activities.

This article then provides arguments and evidence to rethink the exchanges between Swiss and Soviet psychology, focusing on two trips Piaget made to the USSR, first in 1955 when he was invited as President of the IUPSYS to meet the leading figures of Soviet psychology and be informed about their research, and second in 1966 for the 18th International Congress of Psychology in Moscow. The focus is on how Piaget was able to interact and promote scientific exchanges beyond the specific epistemic differences himself and his research team had with their Soviet counterparts. Hence, we highlight Piaget’s role in the international institutionalization of psychology, his organizational skills, and the kind of conditions he favoured for promoting the articulation of psychological research done in different contexts. The analysis of these events shed light on the actual interests of Piaget in Soviet psychology, not just Vygotsky’s theories, and the kind of disciplinary dynamics the Swiss scholar was advocating for beyond specific discussions on cognitive development or socialization. This article is thus part of a series of studies that try to reposition Piaget’s intellectual efforts and biographical events in the light of the institutions he headed and collaborated with, the collective nature of his research, and his role in the international organization of psychology during the 20th century. In this respect, a consideration of Piaget’s endeavours as scientific diplomacy can offer new topics and evidence about his production.

## Piaget and scholars from the socialist bloc: Preliminary experiences

A group of historical works on the relations between Piaget’s and Vygotsky’s programmes assume a decisive asymmetry that marked the interactions: Vygotsky cultivated a relatively deep interest and knowledge on Piaget’s work, while the latter completely ignored Vygotsky until the 1930s and only later, in the 1960s, took a superficial interest and pronounced himself on some of the received critics. Yet there is evidence that Piaget was informed about the readings of his publications by Vygotsky and his colleagues. In 1929 he met Alexander R. Luria, one of Vygotsky’s closest collaborators, during the 9th International Congress of Psychology in Yale, who left a positive impression in him. In a letter, dated 29 October 1929, from Piaget to Ignace Meyerson, who at the time intended to launch a child psychology journal entitled *l’Enfance*, the neuchâtelois suggests the names of those he considered the most prominent authors in the field. Among them, and representing Russia, he mentions ‘Luria, 28 years old; he has many top-level works, he works with Vygotsky, and it will be necessary to include them both’.^1^ Later, in April 1930, Piaget received a signed copy of Vygotsky and Luria’s *Studies on the History of Behaviour: Ape, Primitive and Child*, recently published. While Piaget did not read Russian, this gesture was followed by a prologue to the 1932 Russian translation of *Le langage et la pensée chez l’enfant* (The Language and Thought of the Child), where he mentioned of ‘a series of investigations currently taking place’ which ‘would complement and correct the work made in Geneva’ (Van der Veer, 1996b: 238–9). Thus, Piaget was not completely unaware of the ongoing research of his Russian colleagues.

Despite these initial friendly exchanges, after the Second World War Piaget also had negative experiences in relation to communism. As Latala (2018) shows, during the 1930s Piaget had productive collaborative research with Polish psychologists Maria Zebrowska and Alina Szeminska, but in 1949 it was interrupted by the Zhdanovist rift between socialist and capitalist science and culture. Piaget’s ideas were considered ‘bourgeois’ and thus rejected in Polish academic institutions, and both Zebrowska and Szeminska made a self-critique of their work with Piaget and were no longer allowed to travel to Geneva. The rejection of his work, which in Poland lasted until 1957, surely warned Piaget about the drastic changes in political tides when dealing with psychologists within the socialist countries.

This brief exposition of Piaget’s early exchanges with Eastern European psychologists serve to set precedents for later interactions during the Cold War years, and reconsider some of Piaget objectives in his rapprochement with Soviet scholars.

## Piaget’s first trip to the USSR: A visit to Soviet psychology after Stalinism

The period opened by Stalin’s death in 1953 and the Khrushchev administration allowed Soviet scientists to recover autonomy from the Politburo, reorganize their research, and establish more fluid contact with the Western world (Gerovitch, 2002; Rogacheva, 2016). Psychologists in particular, after being subordinated to Pavlovian Physiology during late Stalinism, were eager to regain disciplinary and political independence to leave behind the Zhdanovist ostracism. In 1954, a group of Soviet experts attended the 14th International Congress of Psychology, held on 7–12 June in Montreal, Canada. This congress was sponsored by the International Union of Scientific Psychology, with two official languages: English and French.^2^ The Soviet delegation was composed by the psychologists Alexei N. Leontiev, Alexander V. Zhaporozhets, and Boris M. Teplov; Grigory S. Kostyuk; and physiologists Evgeny N. Sokolov and Ezras A. Asratian. There, they met Piaget and discussed the possibility of his eventual visit to the USSR. In 1955 Zhaporozhets and Sokolov published a report of the congress in *Voprosy psychologii* (Problems of Psychology), where they refer to Piaget and Inhelder:

Piaget’s lecture, which contained many very illuminating examples to illustrate his arguments, deserves profound study.… As distinct from his former work where he opposed elementary sensory processes to intellectual acts, Piaget has here taken a step forward in the sense that he now recognizes their connection and interaction. Unfortunately, this positive tendency was insufficiently developed in the report, so that it appears that the author still imagines sensory experience and mental operations to be independent factors which only interact in the formation of spatial ideas.… B. Inhelder made an interesting contribution on the development of children’s thinking from 5 to 16. (Zhaporozhets and Sokolov, 1957: 292–3)

These cautious appreciations are meaningful considering that both Swiss authors could easily have been disqualified as ‘bourgeois’ only a couple of years earlier. The report also underlines ‘the efforts of scientists from various countries for the advancement of science and peaceful cooperation between nations’ (ibid.: 295), which shows a renewed intention to restore exchanges with the West.

Piaget’s trip to the USSR finally took place on 24 April 1955 and lasted until 3 May, visiting various research institutions in Moscow and Leningrad. Paul Fraisse, who had taken part in the Montreal Congress, and René Zazzo, a disciple of Henri Wallon and a member of the Communist Party, were also in the same delegation. All three were at that time professors at the Sorbonne’s Institute of Psychology. Henri Piéron and Wallon were also invited, but they finally cancelled their attendance, alleging health reasons. The experience of this visit was documented both by Piaget (1956a, 1956b) and Zazzo (1956).

In his impressions of the Soviet world, Piaget tried to show a picture of the USSR quite different from the mainstream stereotype. For example, regarding partidism, he tried to dissipate doubts about the status of researchers, stressing ‘the position of importance enjoyed in Moscow by men (and women) of science, independent of their position in the party’ (Piaget, 1956a: 393). Moreover, he reproduced an exchange with what he called the ‘five great’ psychologists of the USSR: Alexander Luria, Aleksei Leontiev, Boris Teplov, Sergei Rubinstein, and Anatoli Smirnov. In this respect, he highlighted the possibility of criticism and digression by referring to a ‘diversity of individual opinions on a great number of essential questions, such as, for example, the purpose of psychology’ (ibid.: 393), about which none of the five agreed. In this way, he dispelled suspicions of dogmatic alignment or a climate of intellectual oppression. Another of his remarks aimed to dismantle one installed perception in Western academic circles: ‘We learned *inter alia* that the publications of Ivanov-Smolenski by no means carry authority in Moscow, as is supposed outside the country’ (ibid.: 394). Anatoly G. Ivanov-Smolensky was a psychiatrist trained with Pavlov who later became one of the leading figures in the ‘Pavlovian sessions’ held in the early 1950s, aimed to purge physiology of those researchers not aligned with the Stalinist interpretation of dialectical materialism and Lysenko’s biological theories, and to fully subordinate psychiatry and psychology to a Pavlovian perspective (Windholz, 1997). Considering the dimension of the political intrusion of recent years, Piaget’s remarks do not seem to be naive at all. On the contrary, they seem to show an intention to make it clear that by 1955, Soviet science was regaining autonomy from political intervention. The text ends with a call for scientific dialogue between the USSR and the West, emphasising ‘how anxious the International Union of Scientific Psychology is to be universal’ (Piaget, 1956a: 396), and urging the Soviet Union to publish in foreign languages.

Piaget’s article was first published in French and English in the UNESCO Journal *Bulletin international des sciences sociales* / *International Social Sciences Bulletin* and republished, in English, in the *Newsletter of the International Union of Scientific Psychology*, *American Psychologist*, and *Acta Psychologica*, giving it a significant visibility. However, there was a significative difference in the first version of the article and the rest. In a letter dated 23 March 1956, the then editor of *Acta Psychologica*, H. C. J. Duijker, who was close to IUPSYS, asked Piaget for permission to republish the *Bulletin* article and references of Soviet authors to make contact with.^3^ Piaget accepted the reproduction and asked for three changes: a correction in Luria’s institutional affiliation, the replacement of one erroneous attribution of an experiment, and most importantly, the suppression of the last sentence of this section:

The only critical reflexion that occurred to me in the course of my visit or in analysing certain studies like those of Kostiouk on the psychology of number in children – is that certain writers seem to quote non-Russians a good deal less than they read them. *No doubt there are important reasons for this*. (Piaget, 1956a: 394–5; emphasis added)^4^

Similarly, Filmore H. Sanford, then editor of *American Psychologist*, asked permission to reprint the article, and Piaget replied favourably, though asked for the same changes.^5^ There are two things to consider in this respect; first, the fact that Piaget’s report got published five times show the importance of his account, due to his role as the president of IUPSYS, but also as a reliable version of the situation in the USSR, given that most accounts of the Soviet scenario were considered untrustworthy due to heavy biases and propaganda schemes. Second, in both letters Piaget states that his Moscow hosts agree with the reproduction but asked for those three corrections. The changes are indeed in all versions but the originals. Surely Piaget had some contact with his Soviet counterparts after they got the article from the *Bulletin international des sciences sociales*. On the one hand, their agreement lines up with the intention to make Western connections; on the other, suppression of the last sentence was a gesture of political tact by Piaget towards his fellows, as gratuitous criticism to the Soviet context, even if veiled, could have negative outcomes.

The invitation to Piaget was not isolated; in April of 1955 Brian Simon, a British educator and communist, along with other teachers and educationists, travelled to the USSR and had contact with Luria, Leontiev, and Smirnov, among others, which resulted in a book which compiled articles from Soviet research on education and psychology (Simon, 1957). Later in 1958 a group of pedagogues and psychologists of the American Psychological Association also met with the ‘five greats’ and promised that *American Psychologist* would publish articles from the Eastern Bloc (Murray, May, and Cantril, 1959). At the same time, Soviet psychology publications were reinvigorated, and among new research, some of Vygotsky’s work were republished in two volumes in 1956, which started a sustained effort for making him the leading author of Soviet psychology, both in the USSR and the West (Van der Veer and Yasnitsky, 2016). In this sense, Piaget’s trip was part of a moment of openness of the communist world, after the years of ostracism and post-war Stalinism. In that context, it is possible to consider Piaget’s involvement with Soviet scholars as a form of scientific diplomacy, where the exchanges and collaborations were embedded in wider ideological struggles and the advancement of a discipline acted as a political stance. Political interventions in science have had deleterious results, as Piaget himself experienced, which made disciplinary autonomy not a form of isolation or indifference but a response to authoritarianism and a counterbalance to ideological intolerance.

## Towards the 1966 Moscow Congress

Soon after that first trip in 1955, epistolary exchanges extended relations between Geneva and Moscow. Piaget wrote, among others, to Rubinstein, who was grateful for his ‘kind letter’.^6^ Two years later Rubinstein sent him a handwritten note regretting that he had not been able to find him in Brussels. He also mentioned having no reply from Joseph Nuttin to whom Piaget seems to have spoken about the Soviet psychologist.^7^ For his part, Smirnov accepted Piaget’s article ‘Problèmes de psychologie génétique’ (Problems of Genetic Psychology) for *Voprosy psychologii*, which was translated and published in June 1956.^8^ The journal, edited by Smirnov, Leontiev, Rubinstein, and Teplov, became the leading publication for Soviet psychology during the following three decades. Leontiev also wrote to Piaget shortly after the 1955 meeting, and in February 1957 he informed Piaget of the creation of the Soviet Psychological Society, the first institutional step towards disciplinary and academic autonomy in the USSR. He also informed him of the society’s intention to maintain contact with the IUPSYS, then chaired by Piaget.^9^ Later that year, he invited Piaget to teach a course during 1958 and anticipated that he hoped to see him in April in Italy.^10^ In his reply, Piaget reveals that the meeting did not take place and he proposes to give his course during July, to which Leontiev answers that during that month they have no academic activities, offering him an official invitation for September.^11^ This correspondence demonstrates the common interest of the Soviets in consolidating contacts with the West, and in particular with Piaget, regardless of theoretical or ideological differences.

The exchanges among Vygotsky’s disciples and Piaget continued. In 1959, Leontiev published an article based on his presentation at the Montreal Congress, in which he put forward the idea that Western psychology was changing in a positive direction, highlighting the research of Piaget and his colleagues and placing their ideas on par with what Vygotskian psychologists were working in the USSR (Leontyev, 1981[1959]). Reciprocally, Piaget wrote, a few years later, the text that would be included in the 1962 US edition of Vygotsky’s *Thought and Language*, where he acknowledged the importance of the Soviet psychologist, while, as we said, discussing some of the criticisms that Vygotsky had addressed to him 30 years earlier. That publication was promoted by Luria, but it was one of the translators, Eugenia Hanfmann, who proposed to Piaget to write a reply to Vygotsky’s criticisms of his ideas. We would like to make three considerations about this text. First, Piaget emphasises his link with the Vygotskian school by stating that ‘my friend A. Luria kept me up-to-date concerning Vygotsky’s sympathetic and yet critical position with respect to my own work’ (Piaget, 2000[1962]: 241); second, he regretted his choice of the term ‘egocentrism’ and the misunderstandings he felt it had created; and third he firmly opposed any attempt to determine cognitive development only by its socialization. For him, true cooperative thinking requires the overcoming of both egocentrism and socio-centrism. But these ‘misunderstandings’ and theoretical differences, which were the basis for the Piaget–Vygotsky contraposition mentioned above, cannot overshadow that this text acts as a specific gesture: Piaget’s recognition of Vygotsky as part of a sequence of exchanges that started in 1954, leading to a renewed international recognition of Soviet psychology, the resuming of exchanges disrupted by political dogmatism, and Piaget’s own positioning as promoter and organizer of scholarly networks through constructive criticism.

The book itself was a political stance; the cover had the author’s name in both Latin and Cyrillic alphabets, Jerome Bruner wrote a preface in tune with the ongoing critique of behaviourism of the 1950s and 1960s and, most importantly, promoting a scientific discourse of peaceful and constrictive exchange and competence between the two main global powers. Together with Piaget’s rejoinder, this publication represented productive scholarly dialogue in times of international political intrigues, nuclear secrecy, and military race.

## Piaget’s second trip to the USSR: Lectures at the Kremlin

In 1966 Luria announced the 18th International Congress of Psychology in Moscow. Of the ‘five greats’, Teplov and Rubinstein had passed away. The remaining were in charge of the congress: Leontiev, as president, himself in charge of the programme, and Smirnov responsible for the organization (Luria, 1966). The previous contacts were fruitful: Piaget occupied a more prominent position than any other non-Soviet scholar: he spoke at the opening lectures, took part in a symposium organized by Inhelder and Galperin, and together with Inhelder gave keynotes at the closing event. Piaget also got the support of the Swiss interior minister, Hans-Peter Tschudi, who designated him as Official Swiss delegate.^12^ It was clear it was not a typical scientific meeting.

In fact, it was a massive congress for its time and place, contrasting with previous events: 3897 attendees from 43 countries, it had the political support of the highest Soviet scientific and educational authorities and of the city of Moscow, and the opening session was held in the Kremlin Congress Palace, a recent building made for large PCUS reunions. Nearly 900 lectures were published in at least 35 volumes in Russian, English, and French; there was simultaneous translation for these three languages, two days of film screenings on psychology, and visits to different psychological and neurophysiological laboratories (American Psychological Association, 1968; Rosenzweig *et al.*, 2005). In short, every effort was made to promote Soviet psychology.

It was clear that psychology in the USSR was in a period of renewal. Leontiev’s opening conference, entitled ‘The Concept of Reflex: Its Importance for Scientific Psychology’, interestingly, makes no mention of Pavlov or Lenin. Smirnov’s speech was a recapitulation of almost 50 years of Soviet psychology, where Vygotsky was a ‘young and talented scholar’ whose central aim was to align psychology with Marxism (Smirnov, 1969: 100). Piaget’s lecture was based on the two chapters Piaget had written in April for UNESCO’s *Étude internationale sur les tendances principales de la recherche dans les sciences de l’homme* (International Study on the Main Research Tendencies in the Human Sciences; Piaget, 1966a, 1966b). He lamented that psychology was somewhat stuck between the natural sciences, which had good interdisciplinary relations yet did not include psychology, and the social sciences and humanities, where interdisciplinarity was rare. He proposed a non-linear classification of sciences, to move away from the usual idea of science hierarchies, towards a more cooperative approach between disciplines: ‘The relations between the sciences are therefore not single arrows, but double arrows, in other words circular or spiral relations, which also adjusts to a dialectical spirit’ (Piaget, 1969: 150). The US behaviourist Neal E. Miller closed the evening lectures with a comment on his research on neurophysiological conditioning and psychopathology.

It’s hard not to notice that the three lectures form a picture of diplomatic science during the Cold War years, in particular, Piaget being the mediation between both representatives of the two main global powers, but also between natural and social approaches to psychology. Here is where the Geneva School, coming from a city known for being a centre of international diplomacy, could adopt the role of mediators and promoters of *convivialité*.

Inhelder and Smirnov’s symposium, ‘Psychologie de la formation du concept et des activités mentales’ (The Psychology of Concept Formation and Mental Activity), belonged to a special group of symposia devoted to child development. It included lectures by Piaget, Jerome Bruner, Inhelder, Galperin and François Bresson, and 20 texts were presented for discussion, of which 11 were by Soviet authors, 4 by American authors, and 5 by authors from different Eastern Bloc countries. The closing session featured a presentation by Inhelder. First of all, she underlined the importance of developmental psychology, defining a canon by referring to ‘the heroic times of child psychology, the great masters Vygotsky, Wallon, Bühler, Werner and the young Piaget of the 1920s’ (Inhelder, 1969: 193). Then Inhelder also made incisive remarks in discussing the notion of activity, pointing out the conceptual overlaps between the Genevan programme and Marx’s (well-understood) thought:

We cannot hide our surprise at seeing that, in the society that made possible the invention of the Sputnik, knowledge is considered essentially as a reflection of reality – a conception still close to the empiricism of the 19th century – in which some of the great inspirers of Soviet thought grew up. Let us recall a fundamental aspect of Marx’s thought, that is, that knowledge results from the active interaction of the subject in the transformation of reality: ‘By thus acting on the external world and changing it, he (man) at the same time changes his own nature’. (ibid.: 197)

This provocative statement admits several interpretations but certainly makes clear that the work being done at the CIEG in Geneva was not incompatible with Marxism, and therefore, there was a key intellectual starting point for dialogue and collaboration. Piaget went from being targeted by communist orthodoxies, to promote exchanges between East and West. This renewed legitimacy in the Soviet milieu resulted in several new translations into Russian, including *La genèse des structures logiques élémentaires* (The Genesis of Elementary Logic Sturctures), published in 1963, with a postface by Leontiev and Oleg K. Tikhomirnov; a compilation of *La genèse du nombre chez l’enfant* (The Child’s Conception of Number), *La psychologie de l’intelligence* (The Psychology of Intelligence), and *Logic and Psychology*, published in 1969; and *Traité de psychologie expérimentale* (*Treatise of Experimental Psychology*), published in three volumes, two in 1966 and the last one 1970. Piaget’s reluctance to engage in partisan politics and ideological activism did not imply that his theories could not be considered legitimate among Marxists. In this respect, while his ideas did not feature an apparent political footing, such as the case of his Soviet counterparts, his actions in an institutional level were clearly political, fostering international rapprochement through disciplinary development. This diplomatic disposition was coherent with the international and interdisciplinary research model he was building at the CIEG (Ratcliff, 2019). Piaget’s image as an ascetic rationalist built from his main publications is thus misleading in respect of his attitude towards his engagement with real conditions of research and the value he gave to knowledge production through cooperation.

## Conclusions

Contrary to the assumption that considers Piaget’s response to *Thought and Language* the beginning of asynchronous and dislocated debate, a historical reconstruction of the contacts between Geneva and Moscow allows for the recognition of a longer period in which bonds, references and collaborations were consolidated, and where the exchange of ideas served other purposes than theoretical contraposition. Far from mutual uncommunication or ignorance, Soviet psychologists were quite interested in Piaget’s work, and reciprocally, Piaget was enthusiastic about their research as the School of Geneva advanced to convergences with a dialectically based psychology that could be compatible with Marxist frameworks.

These cross-readings and conceptual debates were consolidated on an organizational level, through a series of collaborations and exchanges, both written and face to face, personal and institutional, which reveal a process of cooperation usually overlooked. In particular, the Cold War conjuncture changed the dynamic of the initial experiences of Piaget with researchers from the socialist bloc, and allowed for both Swiss and Soviet psychologists to have a shared goal, disciplinary autonomy, despite different reasons and conditions: Piaget rejected the subordination of psychology to other disciplines, specially non-empirical philosophy, promoted the international organization of professionals and researchers in psychology, and knew the consequences of political censorship in science; the Soviets reacted to decades of long subordination to physiology and the authoritarian intrusions of the Politburo.

Certainly, Piaget’s prestige among his contemporaries was not only tied to sophisticated theorization, but also to a resourceful organizer with important institutional positions. Piaget embodied part of the Swiss traditional stance of neutrality in world politics, and many times showed himself endorsing the ideal of value neutrality of science. But, even if Piaget tried to embrace such ideal for the sake of objectivity in research, this does not imply that he was neutral in institutional or political terms when it came to psychology as a discipline. Both Piaget and the Soviet psychologists were able to be supported by their political authorities for the 1966 Congress, but it cannot be stated that there was a policy set out ‘from above’ or that both parts were direct representatives of their states. Yet, given the Cold War scenario, those exchanges were necessarily politicized, and the actors involved, aware of this, showed this cooperation as a form of rational rapprochement. It was the antagonism between the ‘Socialist Bloc’ and the ‘Free World’ that made possible to give such exchanges novelty and relevance both scientifically and politically, and the scientific diplomacy of Piaget is shown in the way he articulated his institutional positions to balance between the promotion of international psychological research and the political sensibilities of the time.

## Notes

A previous version of this article was presented in the 54th Annual Meeting of CHEIRON, the International Society for the History of Behavioral and Social Sciences, 21–3 June 2022. We appreciate the reviewers’ suggestions for improving the manuscript.

1. J. Piaget to I. Meyerson, Archives Nationales, Pierrefitte-sur-Seine, AN 521 AP 57. All translations are by the authors unless otherwise stated.

2. ‘14th International Congress of Psychology (Montreal, 7th till 12th June 1954)’, NATO Archives, AC/52-D/39, available at: https://archives.nato.int/14th-international-congress-of-psychology-montreal-7th-till-12th-june-1954.

3. H. C. J. Duijker to J. Piaget, 23 March 1956, Centre Jean Piaget, Université de Genève (hereafter ‘CJP, UNIGE’), AJP/dpf034990.

4. J. Piaget to H. C. J. Duijker, 16 April 1956, CJP, UNIGE, AJP CLA 1956/5.

5. J. Piaget to F. H. Sanford, 16 April 1956, CJP, UNIGE, AJP CLA 1956/5/eng. In Piaget’s replies to Duijker and Sanford, he refers to an English manuscript where that line can be read as ‘cite less foreigners than they read them, but no doubt there are reasons for this over which they have no control’. It seems that Piaget’s English manuscript was different to the version published in the *International Social Sciences Bulletin*, yet it was the latter that was reprinted later, just without the shortened sentence ‘No doubt there are important reasons for this.’ This line in the *Bulletin international des sciences sociales* version was ‘Mais sans doute y a-t-il à cela des raisons indépendantes de leur volonté’ (Piaget, 1956b: 403), which matches the one in the English manuscript Piaget alluded to in the letters.

6. S. Rubinstein to J. Piaget, 17 June 1955, CJP, UNIGE, AJP/dfp030878.

7. S. Rubinstein to J. Piaget, 24 July 1957, CJP, UNIGE, AJP/dfp041935.

8. A. Smirnov to J. Piaget, February 1956, CJP, UNIGE AJP/dfp034991.

9. A. Leontiev to J. Piaget, 26 February 1957, CJP, UNIGE AJP/dfp032147.

10. A. Leontiev to J. Piaget, 28 December 1957, CJP, UNIGE AJP/dfp034982.

11. A. Leontiev to J. Piaget, 22 July 1958, CJP, UNIGE AJP/dfp031230.

12. Designation from Département Fédéral de l’Intérieur to J. Piaget, n.d., CJP, UNIGE AJP/dfp069835.
